# PD-1, PD-L1 and PD-L2 Gene Expression on T-Cells and Natural Killer Cells Declines in Conjunction with a Reduction in PD-1 Protein during the Intensive Phase of Tuberculosis Treatment

**DOI:** 10.1371/journal.pone.0137646

**Published:** 2015-09-11

**Authors:** Syeda S. Hassan, Muhammad Akram, Elizabeth C. King, Hazel M. Dockrell, Jacqueline M. Cliff

**Affiliations:** 1 Department of Immunology and Infection, London School of Hygiene & Tropical Medicine, London, WC1E 7HT, United Kingdom; 2 Gulab Devi Chest Hospital, Lahore, Pakistan; University of Palermo, ITALY

## Abstract

**Background:**

The PD-1 axis is a cell intrinsic immunoregulatory pathway that mediates T cell exhaustion in chronic infection particularly in some viral infections. We hypothesized that PD-1, PD-L1 and PD-L2 would be highly expressed in untreated tuberculosis patients compared to controls due to their chronic infection and would decrease with successful TB treatment.

**Materials and Methods:**

Untreated tuberculosis patients (n = 26) were recruited at diagnosis and followed up during treatment. Household contacts (n = 24) were recruited to establish baseline differences. Blood gene expression *ex vivo* was investigated using qRT-PCR. Flow cytometry was performed to establish protein expression patterns.

**Results:**

PD-L1 gene expression was found to be elevated in active TB disease; however, this was not observed for PD-1 or PD-L2. The intensive phase of TB treatment was associated with a significant decline in PD-1, PD-L1 and PD-L2 gene expression. PD-1 protein expression on the surface of NK cells, CD8^+^ and CD4^+^ T cells was similar in patients with active TB disease compared to controls but declined with successful TB treatment, with the greatest decline occurring on the NK cells followed by CD8^+^ T cells and then CD4^+^ T cells. Granzyme B/PD-1 co-expression declined with successful intensive phase treatment.

**Conclusion:**

Modulation of PD-1/PD-L1 pathway through TB treatment indicates changes in the peripheral T cell response caused by live *Mycobacterium tuberculosis* (*Mtb*) followed by the response to dead bacilli, antigen-release and immuno-pathology resolution. The PD-1 axis could be a host drug target for immunomodulatory treatments in the future.

## Introduction

Tuberculosis (TB) remains a major global health issue as 9 million new cases occurred in 2013 and 1.5 million people succumbed to the disease [1]. The immune system fails to maintain *Mtb* in a latent form in only 5–10% of infected individuals resulting in active disease and pathology [2].

The immune system protects the host by combating *Mtb* but it also has to regulate this response to curtail tissue damage. The cell-intrinsic PD-1 inhibitory pathway is a critical regulatory mechanism which plays an essential role in maintaining the homeostatic balance between positive and negative effects of T cell mediated immunity. In human chronic infections, such as with hepatitis C virus (HCV) and Human Immunodeficiency Virus (HIV), this pathway mediates T cell exhaustion [3–6]. PD-1 and its ligands, PD-L1 (also known as CD274 and B7-H1) and PD-L2 (also known as CD273 and B7-DC), are type I transmembrane proteins. PD-1 is upregulated on T cells upon activation and it exerts its functions only in the presence of antigen-receptor signalling. In humans, PD-L1 has broad expression, including heart, skeletal muscle, placenta and lung tissues and resting myeloid cells [7]. Its expression is inducible on T cells and it is upregulated in many human tumours where it contributes to resistance to CTL-mediated killing [8]. Expression of PD-L1 is also broad on murine T cells and myeloid cells [9]. PD-L2 has a more limited expression pattern, and is inducibly expressed on murine myeloid cells, mast cells and some B cells upon stimulation [9]. In humans, PD-L2 is expressed on dendritic cells [10] and is upregulated on haematopoietic cells by antigen stimulation and Interferons [11]. PD-1 inhibits the TCR signal and can cause IL-2 withdrawal resulting in apoptosis, although this inhibition can be overcome by IL-2 and CD28 [12].

PD-1 and its role in T cell exhaustion was initially determined in microarray studies that looked at gene expression of murine CD8^+^ T cells in lymphocytic choriomeningitis virus (LCMV) infection [13]. In murine studies it has been demonstrated that PD-1 cooperates with other immune exhaustion factors such as T-cell immunoglobulin domain and mucin domain 3 (Tim-3) in chronic viral infections to cause more severe CD8^+^ T cell dysfunction [14]. Virus-specific CD4^+^ T cells in mice also undergo exhaustion but less is known about the mechanism in this cell type [14]. A recent study has demonstrated that re-expanded murine CD8^+^T cells maintain an imprint of epigenetic exhaustion including high PD-1 expression even after chronic infection is resolved [15].

The number of CD3^+^PD-1^+^ cells in the blood and pleural fluid of TB patients is elevated and this correlates positively with IFN-γ production setting up a positive feedback loop [16]. Blockade of the PD-1 axis enhances CD8^+^ T cell cytotoxicity and degranulation [16]. In severe tuberculosis in rhesus monkeys, PD-1 and PD-L2 were upregulated in lymphocytes from the lungs, lymph nodes and spleens [17]. By using siRNA and neutralising antibodies it was demonstrated that PD-1 and cytokine inducible SH2-containing protein (CISH) are necessary for the development of functional regulatory T cells (Tregs) that produce immunomodulatory cytokines such as IL-10 and TGF-β in humans during TB infection [18]. T follicular helper cell distribution and function has also shown to be compromised in active TB, the reason for this was demonstrated to be CTLA-4, PD-L1 and IL-10 expression *in vitro* [19].

The aim of this study was to determine the role of PD-1 and its ligands in human TB and during TB treatment. We hypothesized that PD-1, PD-L1 and PD-L2 would be highly expressed in untreated TB patients compared to latently infected or uninfected contacts due to the chronicity of infection and the regulatory mechanisms invoked to curtail tissue damage. In addition, we hypothesized that a decrease in PD-1, PD-L1 and PD-L2 would be associated with successful treatment due to the decreased bacillary burden lowering the need for immunoregulation, and an accompanying readjustment of the stimulatory/regulatory T cell balance.

## Materials and Methods

### Ethics Statement

This study was approved by the LSHTM (study number 5459) and Gulab Devi Ethics Committees. Informed written consent was obtained from all the study participants prior to obtaining blood samples, investigation of immune responses and sample storage. The study was conducted according to the Declaration of Helsinki principles.

### Subjects and venous blood sample collection

TB patients and household contacts were recruited in a longitudinal study, conducted at Gulab Devi Chest Hospital, Lahore, Pakistan in 2009/2010. Detailed characteristics of subjects, treatment regimen/outcome and recruitment procedures can be found in Table 1 and have been described previously [20]. The TB patients (n = 26) were recruited prior to starting TB treatment and after two months intensive phase treatment according to the National Tuberculosis Program guidelines (2HRZE). It was only possible to follow up a subset of those patients studied pre-treatment at two months due to early discharge of patients from the hospital. Household contact was defined as living in the same room when the TB patient was diagnosed. QuantiFERON-TB Gold (Cellestis, Ltd, Carnegie, Australia) was used in conjunction with lack of clinical symptoms or radiological evidence of TB to categorise the healthy household contacts as latently infected or not infected with *M*. *tuberculosis*.

**Table 1:** Study subjects:clinical and demographic information.

	Age, years: Median [range]	Gender:n, (%) female	TB severity (radiological)	Study follow-up samples	Relationship to TB patient	Latency status (Diagnosis)
TB patients (n = 26)	38 [20–60]	5 (20%)	Moderate:23 Mild:3	2 months (n = 18)	N/A	N/A
Household contacts (n = 24)	40 [20–65]	15 (62%)	N/A	N/A	Spouse = 5 Parent = 10 Child = 2 Sibling = 7	Latently infected = 13 (54%) Not infected = 11 (46%)

Three ml venous blood from each patient or contact was collected into Tempus RNA tubes (Applied Biosystems) and stabilised by shaking vigorously for 10 seconds prior to freezing at -80°C.

### PBMC isolation and freezing

PBMCs were separated from whole blood using density centrifugation over Ficoll-Histopaque 1077 (Sigma-Aldrich, Dorset, U.K.). PBMC were isolated and stimulated immediately as described below, or frozen for downstream flow cytometry using a cryoprotective reagent consisting of 10% DMSO (Sigma-Aldrich, Dorset, U.K.) in AB-Serum-supplemented growth medium. PBMC isolation and freezing were performed in the Microbiology laboratory at Gulab Devi Chest Hospital, Pakistan. Molecular work and flow cytometry were performed in the Immunology laboratories at the London School of Hygiene and Tropical Medicine (London, United Kingdom).

### RNA sample collection and qRT-PCR

RNA was extracted using the Tempus Spin extraction kit (Applied Biosystems) following manufacturer’s instructions. Any potentially contaminating DNA was removed by DNAse treatment, using on-column DNAse (Qiagen) where possible or alternatively *Ambion TURBO* DNA-*free*™ (Invitrogen). RNA quality was checked using a 2100 Bioanalyzer (Agilent Technologies) and was quantified using the Ribogreen assay (Invitrogen).

Equivalent amounts (10 ng) of total RNA from each sample were reverse transcribed using oligo-dT primers and Superscript III (Invitrogen) in 20μl reaction volumes with the total volume diluted to 100 μl prior to downstream use. Quantitative RT-PCR (qRT-PCR) was performed with 5 μl cDNA using either an ABI Prism 7000 or 7500 Fast machine, and Applied Biosystems SYBRGreen reagents. Primers were designed using Primer3 software [21] and gene-specificity checked by nucleotide BLAST [5]: qRT-PCR primer sequence were PD-1 (sense) cgtggcctatccactcctca; PD-1 (anti-sense) atcccttgtcccagccactc; PD-L1 (sense) aaatggaacctggcgaaagc; PD-L1 (anti-sense) gatgagcccctcaggcattt; PD-L2 (sense) gtcttgggagccagggtgac; PD-L2 (anti-sense) tgaaaagtgcaaatggcaagc; Cyclophilin A-S: GCTGGACCCAACACAAATGG; Cyclophilin A-A: TTGCCAAACACCACATGCTT. The gene expression results were normalised to Cyclophilin A. In previous studies of stability of housekeeping genes in human blood samples *ex vivo* and following *in vitro* stimulation, Cyclophilin A was found to be stably expressed (Cliff *et al*, submitted for publication).

### Flow cytometry

Frozen PBMC from study subjects were thawed by drop-wise addition of cold RPMI-1640 medium followed by centrifugation and two washes with RPMI-1640 medium prior to cell counting. The PBMCs were incubated overnight in growth medium at 37°C/5% CO_2_, with Brefeldin A (Becton Dickinson-BD) included for the last three hours. The viability and recovery of cells was between 90–97%. Single stain compensation tubes and fluorescence minus one (FMO) controls were set up. One million PBMCs per tube were stained with anti-CD3-PERCP-Cy5.5 mAb (clone OKT3, e-Bioscience), anti-CD8-FITC mAb (clone OKT8, e-Bioscience) and anti-CD279 (PD-1)-PE mAb (clone MIH4, BD) prior to adding fixation and permeabilisation solutions (BD). Intracellular staining with a pre-titrated amount of anti-Granzyme B-Alexa-Fluor 647 mAb (clone GB-11, BD) was performed. 10% Paraformaldehyde/PBS (Sigma) was added to the samples prior to running on a FACS Calibur 4CA E5180 (BD) under ACDP Biosafety Category 3 containment conditions. The samples were analysed using FlowJo (version 7.6). Lymphocytes were gated using forward scatter against side scatter.

### Statistical analysis

Non-parametric tests in GraphPad Prism were used to analyse the data. The Wilcoxon signed rank test was used for paired data (longitudinal analysis of treatment responses) and the Kruskal-Wallis test with Dunn’s multiple comparison test correction was used to compare the three subject groups: TB patients, latently-infected contacts and non-infected contacts. Receiver Operator Characteristics (ROC) were calculated in GraphPad Prism.

## Results

### PD-1/PD-L2 *ex vivo* gene expression is unaltered whereas PD-L1 expression is elevated in active TB

**Fig 1:**
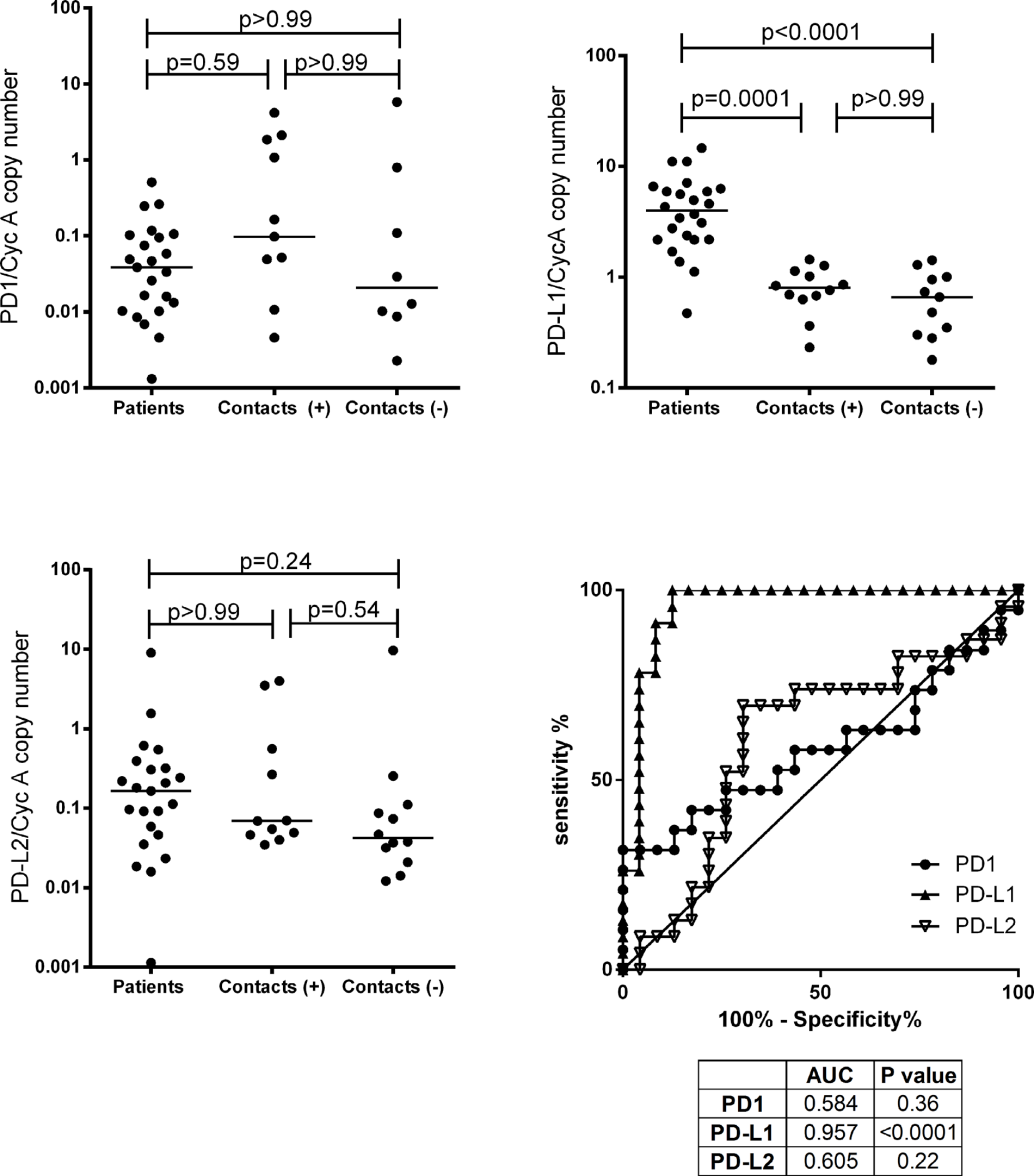
*Ex vivo* gene expression of PD-1 and its ligands in active TB disease. RNA was extracted from whole blood samples added to Tempus tubes and expression investigated by qRT-PCR. (A) PD-1 expression in *ex vivo* venous blood from untreated active TB cases at diagnosis (n = 23), in IGRA+ (C+: n = 11) and in IGRA- contacts (C-: n = 8). (B) PD-L1 expression in *ex vivo* venous blood from untreated active TB cases at diagnosis (n = 24), in latently-infected household contacts (C+: n = 12) and in uninfected contacts (C-: n = 11). (C) PD-L2 expression in *ex vivo* venous blood from untreated active TB cases at diagnosis (n = 24), in latently-infected household contacts (C+: n = 12) and in uninfected contacts (C-: n = 12). The lines in the centre of each group represent medians. (D) ROC comparisons were conducted between TB patients and household contacts (C+ and C- combined) to determine whether PD-1, PD-L1 or PD-L2 expression could distinguish active TB patients from healthy people. PD-1/PD-L1/PD-L2 mRNA expression was determined by qRT-PCR, with results shown normalised against the housekeeping gene Cyclophilin A. P values in A) to C) were derived from Kruskal-Wallis test with Dunn’s multiple testing correction. AUC = Area Under the Curve.

We first sought to investigate whether PD-1, PD-L1 and PD-L2 gene expression in peripheral blood was elevated during active TB disease. The expression of PD-1 was measured in *ex vivo* RNA samples from TB patients and from latently infected and non-infected contacts from Pakistan. Contrary to our expectation that PD-1, being immunoregulatory, would be elevated in active TB disease due to establishment of chronic disease, no significant differences were observed in PD-1 gene expression between untreated TB patients, latently infected contacts and uninfected contacts at diagnosis (Fig 1A).

To better understand the role of this axis, it was important to look at PD-1 ligand expression as PD-1 binds to PD-L1 or PD-L2 in order to exert its suppressive effects on T cell proliferation, cytotoxicity and cytokine protection. PD-L1 expression in blood was significantly higher in TB patients at diagnosis compared to both latently infected (p = 0.0001) and uninfected contacts (p<0.0001) (median normalised PD-L1 expression; TB patients = 5 arbitrary units; latently infected = 0.7 arbitrary units and uninfected contacts = 0.7 arbitrary units) (Fig 1B). PD-L1 expression amongst the two groups of contacts with or without latent TB infection (LTBI) was similar. Active TB patients at diagnosis had similar PD-L2 levels compared to latently infected (p = 0.8) and uninfected contacts (p = 0.09) (Fig 1C). To further analyse whether expression PD-1 or its ligands could discriminate TB patients from household contacts, ROC curves were analysed: PD-L1 expression had an AUC of 0.957 with P<0.0001, whereas the ROC curves for PD-1 and PD-L2 were again non-significant (Fig 1D). Thus *ex vivo* PD-1/PD-L2 gene expression was unaltered by active disease whereas PD-L1 gene expression was elevated.

### PD-1 and PD-L1 gene expression is negatively modulated during the intensive phase of TB treatment whereas PD-L2 shows a differing pattern

**Fig 2:**
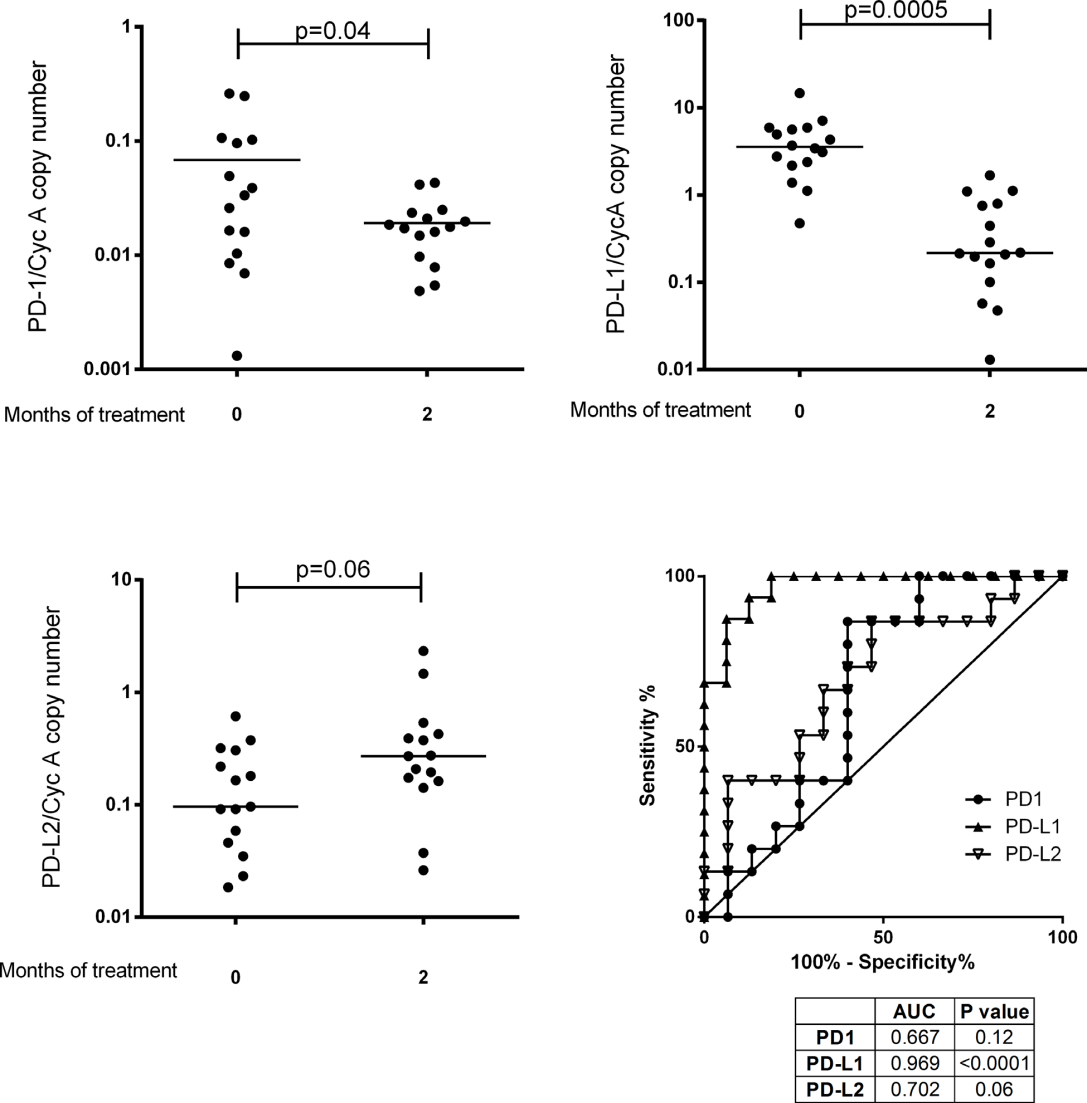
*Ex vivo* gene expression of PD-L1 and PD-L2 in the intensive phase of TB treatment. RNA was extracted from whole blood added to Tempus tubes and expression investigated by qRT-PCR. (A) PD-1 expression at the beginning and the end of the intensive phase (n = 15 patients). (B) PD-L1 expression at the beginning and the end of the intensive phase (n = 16 patients). (C) PD-L2 expression at the beginning and the end of the intensive phase (n = 15 patients). The lines in the centre of each group represent medians. (D) ROC comparisons were conducted for PD-1, PD-L1 and PD-L2 expression comparising TB diagnosis with month 2 of treatment for the TB patients. PD-L1/PD-L2 mRNA expression was normalised against the housekeeping gene Cyclophilin A.

The TB patients recruited at diagnosis were followed longitudinally during TB treatment. The intensive phase of chemotherapy led to a decline in PD-1 gene expression (p = 0.04) (Fig 2A). An even more marked decline was observed in the case of PD-L1 (p = 0.0005) (Fig 2B). Down-regulation of PD-1 and PD-L1 gene expression is indicative of a decrease in cell exhaustion, likely to be due to the lowered *Mtb* bacillary burden brought about by successful treatment during the intensive phase of chemotherapy. There was weak statistical evidence for chemotherapy elevating PD-L2 gene expression (p = 0.06) (Fig 2C). ROC analysis showed that the changes in expression of PD-L1 in TB patients had a greater capacity to distinguish TB diagnosis and 2-month treatment time points than PD-1 or PD-L2 (Fig 2D). This differing pattern of gene expression could be due to the differing tissue expression patterns of PD-L1 and PD-L2 with the former mediating PD-1 signalling on T cells and APCs and with the latter affecting expression on macrophages and dendritic cells.

### The frequency of PD-1 expressingCD4 T^+^ cells, CD8^+^ T cells and NK cells is down-regulated by the intensive phase of TB treatment

**Fig 3:**
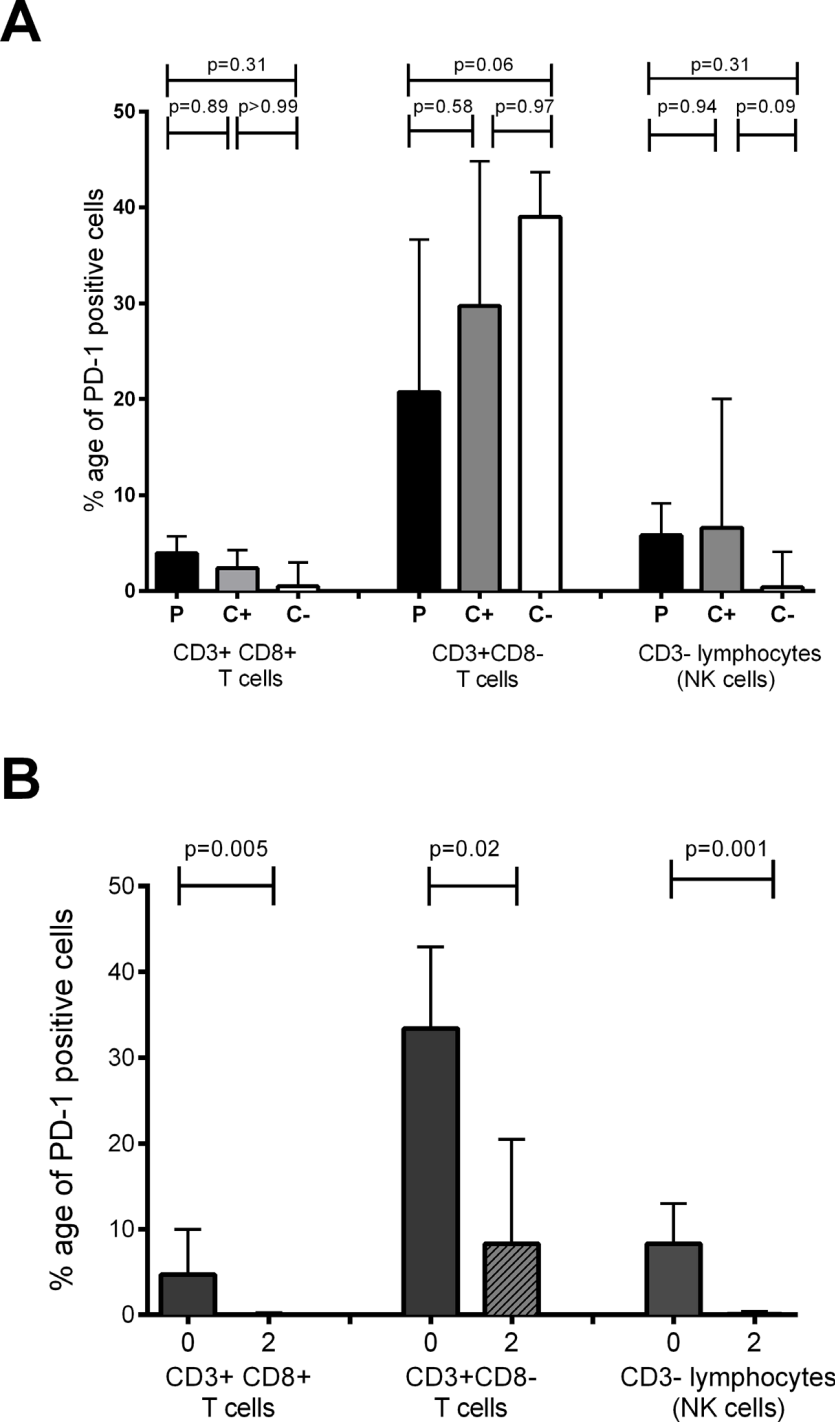
PD-1 protein expression during active TB disease and chemotherapy. Frozen PBMCs from Pakistan were thawed and stained to analyse PD-1 protein expression. (A) PD-1 protein expression on CD3^+^CD8^+^ T cells, CD3^+^CD8^-^ T cells and CD3^-^ lymphocytes at diagnosis in 18 TB patients (moderate severity) (P), 7 latently infected contacts (C+) and 5 uninfected contacts (C-). P values are derived from the Kruskal-Wallis test with Dunn’s multiple testing correction. (B) Modulation of PD-1 protein expression on CD3^+^CD8^+^ T cells, CD3^+^CD8^-^ T cells and CD3^-^ lymphocytes with the intensive phase of chemotherapy in patients who were sputum smear negative after 2 months of treatment (n = 11), analysed with the Wilcoxon signrank test. The bars show medians and the interquartile range.

As PD-1 mRNA expression was modulated by TB treatment, we next investigated whether these differences were translated into differences in protein expression. Vitality of cryopreserved PBMC was assured by live/dead staining superfluous samples (not shown). Stained PBMC from TB patients and household contacts were analysed as shown in S1 Fig. No significant differences in the frequency of PD-1 expressing cells were observed at TB diagnosis on CD8^+^ T cells or CD3^-^ lymphocytes (likely NK cells) when TB patients, latently-infected and non-infected household contacts were all compared by the Kruskal-Wallis test. There was weak statistical evidence for a lower frequency of PD-1 expression on CD3^+^CD8^-^ T cells (likely CD4^+^ T cells) from TB patients compared to uninfected household contacts (Fig 3A). The frequency of PD-1 expressing cells was significantly decreased during the first 2 months of TB treatment in both the CD3^+^CD8^+^ T cells (p = 0.005, Wilcoxon ranksum test) and in the non-T cell lymphocyte subset (p = 0.001, Wilcoxon ranksum test) (Fig 3B). This decline was also observed in the CD3^+^CD8^-^ T cell compartment although it was less pronounced than in the CD3^+^CD8^+^ T cells (p = 0.02) (Fig 3B).

### TB treatment lowers the frequency of PD-1+GranzymeB+CD3+CD8- T cells

**Fig 4:**
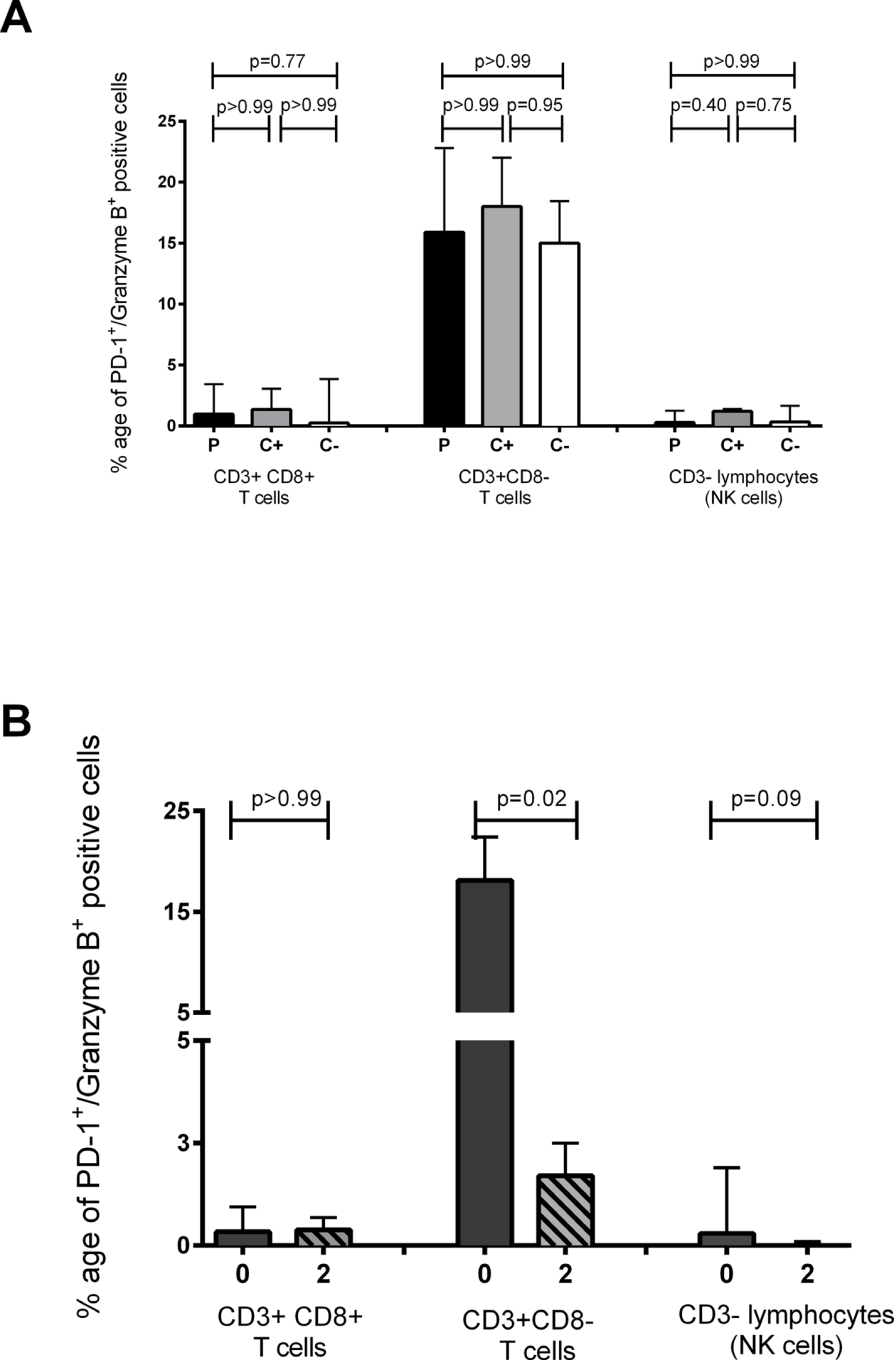
Modulation of PD-1 and GrzB protein co-expression during active TB disease and chemotherapy. Frozen PBMCs from Pakistan were thawed and PD-1 and GrzB co-expression was investigated by flow cytometry. (A) The proportion of PD-1 cells which are also GzB positive in the CD3^+^CD8^+^ lymphocyte gate, CD3^+^CD8^-^ lymphocyte gate and CD3^-^ lymphocyte (NK cell) gate at diagnosis in TB patients (P: n = 18), latently infected contacts (C+: n = 7) and uninfected contacts (C-: n = 4). P values are derived from the Kruskal-Wallis test with Dunn’s multiple testing correction (B) Modulation of PD-1/GzB co-expression with the intensive phase of chemotherapy on CD3^+^CD8^+^ T cells, CD3^+^CD8^-^ lymphocytes and CD3^-^ lymphocytes (NK cells), analysed with the Wilcoxon signrank test. The bars show medians and the interquartile range.

We next investigated PD-1 expression in concert with Granzyme B expression, to establish whether PD-1 has an impact on cytotoxic potential in active TB and during treatment. No significant differences were found between PD-1/Granzyme B co-expression at diagnosis between the untreated TB patients, latently infected and uninfected contacts and this was true for the CD3^+^CD8^+^ T cells, the CD3^-^ lymphocyte subset and the CD3^+^CD8^-^ T cells (Fig 4A). Frequency of PD-1/Granzyme B co-expression remained unaltered in CD8^+^ T cells after the intensive phase of treatment (p>0.99) (Fig 4B). There was a slight but insignificant trend towards decreasing PD-1/Granzyme B co-expression in the non-T cell lymphocyte compartment (p = 0.09) (Fig 4B) although this was a minor cell population. A significant decline in frequency of PD-1/Granzyme B co-expressing cells was observed on CD3^+^CD8^-^ T cells from TB patients after two months of intensive TB chemotherapy (p = 0.02) (Fig 4B).

## Discussion

Our hypothesis was that TB patients would have increased proportions of PD-1^+^CD8^+^ and CD4^+^ T cells and higher PD-1 and associated ligand gene expression compared to household contacts and that chemotherapy would reduce the expression of these molecules allowing for immune recovery. However, we discovered that expression of PD-1, PD-L1 and PD-L2 was similar in TB patients and in latently infected (IGRA-positive) and non-infected household contacts at TB diagnosis. Nonetheless, we found that the gene expression of PD-1 and PD-L1 in whole blood decreased during successful TB treatment, and that the expression of PD-L2 moderately increased. To our knowledge, this is the first study to investigate PD-1 and its ligand expression patterns in an Asian cohort during TB treatment, although a previous microarray-based study on South African and British TB patients reported that PD-L1 expression is elevated on neutrophils in TB disease and decreases with subsequent treatment [22]. By using qRT-PCR, rather than microarray technology which can only show relative changes, we have demonstrated that measurement of gene expression PD-1 and its ligands could be developed into an easy, robust method to monitor TB treatment response. We have also shown that changes in gene expression of PD-1 through treatment are translated into changes in protein expression, particularly on T cells.

PD-L1 *ex vivo* gene expression was elevated at TB diagnosis in patients compared to contacts indicating higher PD-1 ligation on immune cells. PD-L2 *ex vivo* gene expression at diagnosis showed no difference between contacts and TB patients. We expected that PD-1, as a marker of T cell immunoregulation or immune activation, would be higher in TB patients compared to contacts due to the established chronicity of active TB infection in the former group. Surprisingly, PD-1 gene and protein expression on CD8^+^ T cells, non-CD8^+^ T cells (likely CD4^+^ T cells) and non CD3^+^ lymphocytes (likely NK cells) were not found to be significantly different between the patients and their contacts; this could be due to the fact that increased availability of ligand in patients ensures greater downstream signaling of the pathway making up-regulation of receptor expression unnecessary. Future work should include direct flow cytometry stains for CD4 and CD56: their inclusion was prevented in this study by patient cell yield and technology available in the ACDP Biosafety Category 3 Facility. One previous study has shown similar results for PD-1 expression as it was demonstrated that HIV-TB patients showed increased basal levels of PD-1 on T lymphocytes whereas this was not the case for TB patients who did not have HIV co-infection [23]. In contrast, another study has shown that following *Mtb* re-stimulation of PBMC from TB patients *in vitro* CD3^+^ cells expressing PD-1^+^ increase [16]. Another study has also demonstrated that PD-1 expression is higher on CD4^+^ T cells in TB patients compared to controls whereby IL-17 production is decreased, pSTAT3 inhibited and IL-23R expression decreased [24]. These different observations might be due to *in vitro* stimulation rather than direct *ex vivo* protein expression. This study [24], similarly to ours, demonstrated a decrease in PD-1 expression on the surface of CD4^+^ T cells with TB treatment. Another study has similarly shown elevated expression of PD-1 on the surface of B cells, monocytes and T cells in patients with active disease and that chemotherapy leads to negative modulation of the PD-1 pathway [25]. The intensive phase of TB treatment was associated with a decline in PD-1, PD-L1 and PD-L2 gene expression. This could be due to a decrease in frequency of PD-L1 and 2 expressing cells in the blood of cured TB patients or decreased PD-L1 and 2 expression by each individual cell. PD-1^+^CD8^+^ T cells declined with successful TB treatment in a manner more pronounced than on CD4^+^ T cells but to a lesser extent than on NK cells. This is indicative of the decreased bacillary burden after the intensive phase of therapy as NK cells and T cells are probably showing signs of recovery from inhibition or dampening of the immune system. Also, this decline in the intensive phase of treatment could be due to mycobacterial cell death resulting in release of antigen followed by pathology resolution by the end of treatment as we have previously demonstrated [20]. It is not entirely clear why there was little difference in PD-1 expression between TB patients at diagnosis and household contacts, and yet there was a significant decrease after two months of TB treatment. One possibility is that because TB patients exhibit enhanced apoptosis in their PD-1^+^ CD4^+^ T cell compartment [26], this cell population disappeared from the circulation during treatment as antigenic stimulation was removed, and would have taken a longer time period to be replenished. Similar rapid drops in PD-1 expression on CD4^+^ and CD8^+^ T cells in *M*. *tuberculosis*-infected mouse lungs upon drug treatment have been observed previously [27]. In other TB-treatment biomarker discovery studies [28], transient changes in some cytokine concentrations in blood, such as TNFα, MIP1α/β, IL-10 and sCD40L have been reported, such that changes observed early in TB treatment reverted to levels similar to those seen both pre-treatment and in healthy control subjects. Further experiments with longer follow-up and more detailed phenotypic analysis would be required to properly address this issue.

There is a gradient in response showing that NK cells emerge from a state of being responsive to inhibitory signals capable of dampening the immune system first followed by CD8^+^ T cells and finally CD4^+^ T cells which could indicate that cytotoxic potential is restored before cytokine releasing potential. A recent study has evaluated the role of PD-1 and its ligands on NK cells from active TB patients and shown that active TB disease and mycobacterial stimulation leads to higher expression of PD-1/ligands on NK cells leading to inhibition of their effector functions like cytokine production [29]. GrzB/PD-1 co-expression was not altered by active disease or chemotherapy except in the CD4^+^ T cell compartment suggesting that PD-1 might not be suppressing the killing activity of CD8^+^ T cells and NK cells but rather modulating the immune system through another mechanism such as via anti-inflammatory/immunoregulatory cytokine production, impairment of other effector functions and apoptosis. A recent study has identified one such mechanism by which PD-1^+^ NKT cells correlate with bacillary load in newly diagnosed TB patients with treatment leading to a decline in their frequency [30]. In addition, this study demonstrated that PD-1 expression leads to apoptosis of IFN-γ producing NKT cells but not IL-4 producing ones [30]. In mice, PD-1 has been demonstrated to affect CD4^+^ and CD8^+^ T cell effector functions by inhibiting the T-bet, GATA-3 and Eomes transcription factors which are responsible for Th1 lineage commitment, secretion of cytokines from Th2 cells and T cell development respectively [31].

The role of PD-1 and its ligands in murine TB remains controversial. PD-1 positive CD4^+^ T cells are not exhausted in *Mtb* infected mice; instead they retain proliferative capacity and replenish effector cells by differentiating into KLRG-1 positive T cells capable of secreting TNF-α and IFN-γ [32]. PD-1 deficient mice have a larger number of proliferating CD4^+^ T cells in their lungs driving pathology rather than controlling *Mtb* infection [33]. PD-1^-/-^ mice are more susceptible to *Mtb* infection compared to their wild-type counterparts due to the increased frequency of Tregs and reduced T/B cell proliferation, supporting the hypothesis that PD-1 plays a protective role in murine TB [34]. A recent paper has shown that the survival of PD-1 ^-/-^ mice after aerosol infection with *Mtb* is severely reduced compared to wild-type mice due to significantly higher bacterial load and severe multifocal necrotic pneumonia [35]. Our study demonstrates that high levels of PD-L1 expression in untreated human TB may allow infection to be maintained by preventing *Mtb* clearance indicating that the role of this axis may be harmful in human TB. The exact mechanism by which the PD-1 axis exerts its role in human TB still remains to be elucidated and the patterns observed in this study should be confirmed with larger cohorts from different geographical populations.

This study is one of the first to look at PD-1, PD-L1 and PD-L2 gene and protein expression during treatment in humans with pulmonary TB. In conclusion, we have shown here that PDL1/2 gene expression and the frequency of PD-1 expressing T cells and NK cells is modulated in peripheral blood during the intensive phase of successful TB treatment. The extent to which this decline occurs may depend on the role each cell type plays in the TB immune response. The observation that PD-L1 expression is higher in active TB patients compared to latently infected contacts demonstrates that the latter might be protected due to lower PD-1 pathway signaling and decreased T cell immunoregulation as a result of a lower bacillary burden leading to the mounting of a more efficacious immune response against *Mtb* infection. The importance of this pathway in human TB should be explored further as it could be exploited therapeutically enriching the limited arsenal of drugs currently available. The putative biomarkers of TB treatment response tested in this study should be tested in future large-scale biomarker studies.

## Supporting Information

S1 Fig Flow cytometry gating strategy on a representative TB patient PBMC samples. (A) Cells were studied within the lymphocyte gate that was drawn according to forward scatter (FSC) and side scatter (SSC) properties eliminating dead cells, debris and monocytes. (B) CD3 and CD8 expression was analysed within the lymphocyte gate and three populations gated: CD3^+^CD8^-^ cells, CD3^+^CD8^+^ cells and CD3^-^CD8^-^ cells. PD-1 and Granzyme B expression were assessed within CD3^+^CD8^-^ cells (C), CD3^-^CD8^-^ cells (D) and CD3^+^CD8^+^ T cells (E). In panels C, D and E the upper right quadrant shows PD-1^+^GrzB^+^ cells, the upper left quadrant shows PD-1^+^GrzB^-^ cells, the lower right quadrant shows PD1^-^GrzB^+^ cells and the lower left quadrant shows PD-1^-^GrzB^-^ cells. The staining panel comprised anti-CD3 PERCP, anti-CD8 FITC, anti-PD-1 PE and Granzyme B APC. Isotype controls were used for PD-1 and Granzyme B and Fluorescence Minus One Controls (FMOs) were used to set the gates accordingly.
(TIF)

## References

[1] WHO. Global Tuberculosis Report. 2014.

[2] Stewart GR, Robertson BD, Young DB. Tuberculosis: a problem with persistence. Nat Rev Microbiol. 2003;1(2):97–105. Epub 2004/03/24. 10.1038/nrmicro749 . 15035039

[3] Petrovas C, Casazza JP, Brenchley JM, Price DA, Gostick E, Adams WC, et al PD-1 is a regulator of virus-specific CD8+ T cell survival in HIV infection. The Journal of experimental medicine. 2006;203(10):2281–92. Epub 2006/09/07. 10.1084/jem.20061496 16954372 PMC2118095

[4] Trautmann L, Janbazian L, Chomont N, Said EA, Gimmig S, Bessette B, et al Upregulation of PD-1 expression on HIV-specific CD8+ T cells leads to reversible immune dysfunction. Nature medicine. 2006;12(10):1198–202. Epub 2006/08/19. 10.1038/nm1482 . 16917489

[5] Day CL, Kaufmann DE, Kiepiela P, Brown JA, Moodley ES, Reddy S, et al PD-1 expression on HIV-specific T cells is associated with T-cell exhaustion and disease progression. Nature. 2006;443(7109):350–4. . 16921384 10.1038/nature05115

[6] Urbani S, Amadei B, Tola D, Massari M, Schivazappa S, Missale G, et al PD-1 expression in acute hepatitis C virus (HCV) infection is associated with HCV-specific CD8 exhaustion. Journal of virology. 2006;80(22):11398–403. Epub 2006/09/08. 10.1128/jvi.01177-06 16956940 PMC1642188

[7] Dong H, Zhu G, Tamada K, Chen L. B7-H1, a third member of the B7 family, co-stimulates T-cell proliferation and interleukin-10 secretion. Nat Med. 1999;5(12):1365–9. 10.1038/70932 . 10581077

[8] Greaves P, Gribben JG. The role of B7 family molecules in hematologic malignancy. Blood. 2013;121(5):734–44. 10.1182/blood-2012-10-385591 23223433 PMC3563361

[9] Yamazaki T, Akiba H, Iwai H, Matsuda H, Aoki M, Tanno Y, et al Expression of programmed death 1 ligands by murine T cells and APC. J Immunol. 2002;169(10):5538–45. Epub 2002/11/08. . 12421930 10.4049/jimmunol.169.10.5538

[10] Tseng SY, Otsuji M, Gorski K, Huang X, Slansky JE, Pai SI, et al B7-DC, a new dendritic cell molecule with potent costimulatory properties for T cells. J Exp Med. 2001;193(7):839–46. 11283156 10.1084/jem.193.7.839 PMC2193370

[11] Kulpa DA, Lawani M, Cooper A, Peretz Y, Ahlers J, Sekaly RP. PD-1 coinhibitory signals: the link between pathogenesis and protection. Semin Immunol. 2013;25(3):219–27. 10.1016/j.smim.2013.02.002 23548749 PMC3795833

[12] Keir ME, Butte MJ, Freeman GJ, Sharpe AH. PD-1 and its ligands in tolerance and immunity. Annu Rev Immunol. 2008;26:677–704. Epub 2008/01/05. 10.1146/annurev.immunol.26.021607.090331 . 18173375 PMC10637733

[13] Yi JS, Cox MA, Zajac AJ. T-cell exhaustion: characteristics, causes and conversion. Immunology. 2010;129(4):474–81. Epub 2010/03/06. IMM3255 [pii] 10.1111/j.1365-2567.2010.03255.x 20201977 PMC2842494

[14] Jin HT, Anderson AC, Tan WG, West EE, Ha SJ, Araki K, et al Cooperation of Tim-3 and PD-1 in CD8 T-cell exhaustion during chronic viral infection. Proc Natl Acad Sci U S A. 2010;107(33):14733–8. Epub 2010/08/04. 1009731107 [pii] 10.1073/pnas.1009731107 20679213 PMC2930455

[15] Utzschneider DT, Legat A, Fuertes Marraco SA, Carrie L, Luescher I, Speiser DE, et al T cells maintain an exhausted phenotype after antigen withdrawal and population reexpansion. Nature immunology. 2013;14(6):603–10. Epub 2013/05/07. 10.1038/ni.2606 . 23644506

[16] Jurado JO, Alvarez IB, Pasquinelli V, Martinez GJ, Quiroga MF, Abbate E, et al Programmed death (PD)-1:PD-ligand 1/PD-ligand 2 pathway inhibits T cell effector functions during human tuberculosis. J Immunol. 2008;181(1):116–25. . 18566376 10.4049/jimmunol.181.1.116

[17] Qiu L, Huang D, Chen CY, Wang R, Shen L, Shen Y, et al Severe tuberculosis induces unbalanced up-regulation of gene networks and overexpression of IL-22, MIP-1alpha, CCL27, IP-10, CCR4, CCR5, CXCR3, PD1, PDL2, IL-3, IFN-beta, TIM1, and TLR2 but low antigen-specific cellular responses. J Infect Dis. 2008;198(10):1514–9. 10.1086/592448 18811584 PMC2884371

[18] Periasamy S, Dhiman R, Barnes PF, Paidipally P, Tvinnereim A, Bandaru A, et al Programmed death 1 and cytokine inducible SH2-containing protein dependent expansion of regulatory T cells upon stimulation With Mycobacterium tuberculosis. J Infect Dis. 2011;203(9):1256–63. 10.1093/infdis/jir011 21383382 PMC3069733

[19] Kumar NP, Sridhar R, Hanna LE, Banurekha VV, Nutman TB, Babu S. Decreased frequencies of circulating CD4(+) T follicular helper cells associated with diminished plasma IL-21 in active pulmonary tuberculosis. PLoS One. 2014;9(10):e111098 10.1371/journal.pone.0111098 25343703 PMC4208798

[20] Hassan SS, Cho JE, Akram M, Fielding KL, Dockrell HM, Cliff JM. Modulation of NKG2D expression in human CD8(+) T cells corresponding with tuberculosis drug cure. PloS one. 2013;8(7):e70063 Epub 2013/08/08. 10.1371/journal.pone.0070063 23922903 PMC3724721

[21] Rozen S, Skaletsky H. Primer3 on the WWW for general users and for biologist programmers. Methods in molecular biology (Clifton, NJ). 2000;132:365–86. Epub 1999/11/05. . 10547847 10.1385/1-59259-192-2:365

[22] McNab FW, Berry MP, Graham CM, Bloch SA, Oni T, Wilkinson KA, et al Programmed death ligand 1 is over-expressed by neutrophils in the blood of patients with active tuberculosis. Eur J Immunol. 2011;41(7):1941–7. 10.1002/eji.201141421 21509782 PMC3179592

[23] Jurado JO, Pasquinelli V, Alvarez IB, Martinez GJ, Laufer N, Sued O, et al ICOS, SLAM and PD-1 expression and regulation on T lymphocytes reflect the immune dysregulation in patients with HIV-related illness with pulmonary tuberculosis. Journal of the International AIDS Society. 2012;15(2):17428 10.7448/IAS.15.2.17428 22713261 PMC3499801

[24] Bandaru A, Devalraju KP, Paidipally P, Dhiman R, Venkatasubramanian S, Barnes PF, et al Phosphorylated STAT3 and PD-1 regulate IL-17 production and IL-23 receptor expression in Mycobacterium tuberculosis infection. Eur J Immunol. 2014;44(7):2013–24. 10.1002/eji.201343680 24643836 PMC4106993

[25] Singh A, Mohan A, Dey AB, Mitra DK. Inhibiting the programmed death 1 pathway rescues Mycobacterium tuberculosis-specific interferon gamma-producing T cells from apoptosis in patients with pulmonary tuberculosis. J Infect Dis. 2013;208(4):603–15. 10.1093/infdis/jit206 . 23661793

[26] Elliott TO, Owolabi O, Donkor S, Kampmann B, Hill PC, Ottenhoff TH, et al Dysregulation of Apoptosis Is a Risk Factor for Tuberculosis Disease Progression. J Infect Dis. 2015 10.1093/infdis/jiv238 . 25895988

[27] Henao-Tamayo M, Irwin SM, Shang S, Ordway D, Orme IM. T lymphocyte surface expression of exhaustion markers as biomarkers of the efficacy of chemotherapy for tuberculosis. Tuberculosis (Edinb). 2011;91(4):308–13. 10.1016/j.tube.2011.04.001 21530406 PMC3155998

[28] Djoba Siawaya JF, Beyers N, van Helden P, Walzl G. Differential cytokine secretion and early treatment response in patients with pulmonary tuberculosis. Clin Exp Immunol. 2009;156(1):69–77. Epub 2009/02/07. CEI3875 [pii] 10.1111/j.1365-2249.2009.03875.x 19196252 PMC2673743

[29] Alvarez IB, Pasquinelli V, Jurado JO, Abbate E, Musella RM, de la Barrera SS, et al Role played by the programmed death-1-programmed death ligand pathway during innate immunity against Mycobacterium tuberculosis. J Infect Dis. 2010;202(4):524–32. 10.1086/654932 . 20617899

[30] Singh A, Dey AB, Mohan A, Mitra DK. Programmed death-1 receptor suppresses gamma-IFN producing NKT cells in human tuberculosis. Tuberculosis. 2014;94(3):197–206. 10.1016/j.tube.2014.01.005 . 24629634

[31] Nurieva R, Thomas S, Nguyen T, Martin-Orozco N, Wang Y, Kaja MK, et al T-cell tolerance or function is determined by combinatorial costimulatory signals. EMBO J. 2006;25(11):2623–33. Epub 2006/05/26. 7601146 [pii] 10.1038/sj.emboj.7601146 16724117 PMC1478197

[32] Reiley WW, Shafiani S, Wittmer ST, Tucker-Heard G, Moon JJ, Jenkins MK, et al Distinct functions of antigen-specific CD4 T cells during murine Mycobacterium tuberculosis infection. Proc Natl Acad Sci U S A. 2010 Epub 2010/10/22. 1006298107 [pii] 10.1073/pnas.1006298107 . 20962277 PMC2984157

[33] Barber DL, Mayer-Barber KD, Feng CG, Sharpe AH, Sher A. CD4 T cells promote rather than control tuberculosis in the absence of PD-1-mediated inhibition. J Immunol. 2011;186(3):1598–607. Epub 2010/12/22. jimmunol.1003304 [pii] 10.4049/jimmunol.1003304 . 21172867 PMC4059388

[34] Tousif S, Singh Y, Prasad DV, Sharma P, Kaer LV, Das G. T Cells from Programmed Death-1 Deficient Mice Respond Poorly to Mycobacterium tuberculosis Infection. PLoS ONE. 2011;6(5):e19864 Epub 2011/05/19. [pii]. 21589883 10.1371/journal.pone.0019864 PMC3093409

[35] Lazar-Molnar E, Chen B, Sweeney KA, Wang EJ, Liu W, Lin J, et al Programmed death-1 (PD-1)-deficient mice are extraordinarily sensitive to tuberculosis. Proc Natl Acad Sci U S A. 2010;107(30):13402–7. Epub 2010/07/14. 1007394107 [pii] 10.1073/pnas.1007394107 . 20624978 PMC2922129

