# Supplementary figures and images for: PD-1, PD-L1 and PD-L2 Gene Expression on T-Cells and Natural Killer Cells Declines in Conjunction with a Reduction in PD-1 Protein during the Intensive Phase of Tuberculosis Treatment
Source: PLoS One. 2015 Sep 11;10(9):e0137646. doi: 10.1371/journal.pone.0137646 (PMC4567315; doi:10.1371/journal.pone.0137646)

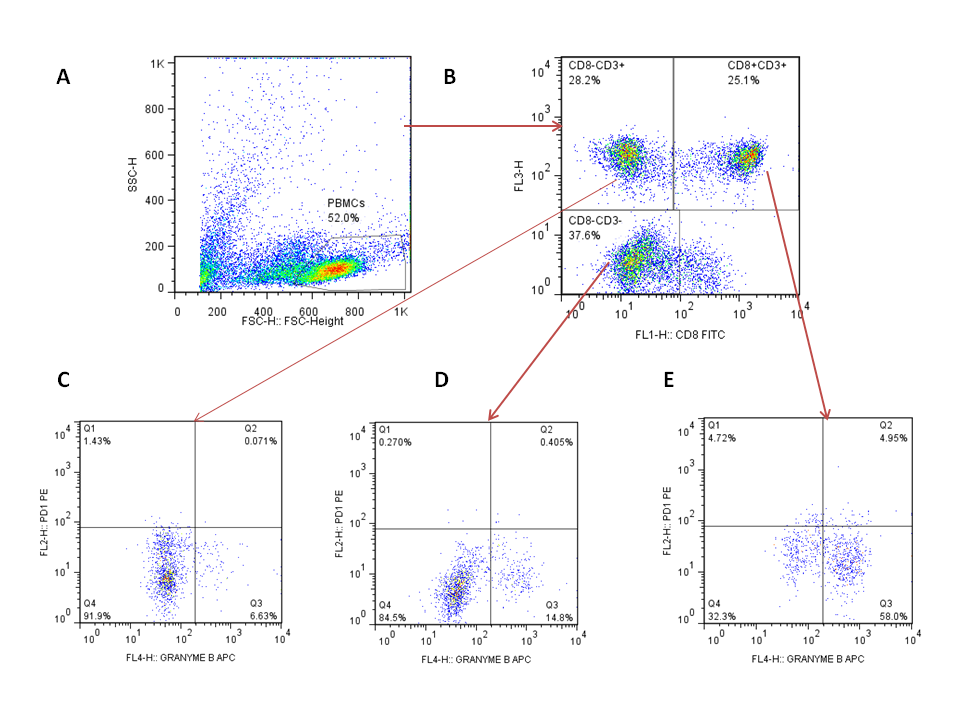

Supplement: S1 Fig — (A) Cells were studied within the lymphocyte gate that was drawn according to forward scatter (FSC) and side scatter (SSC) properties eliminating dead cells, debris and monocytes. (B) CD3 and CD8 expression was analysed within the lymphocyte gate and three populations gated: CD3+CD8- cells, CD3+CD8+ cells and CD3-CD8- cells. PD-1 and Granzyme B expression were assessed within CD3+CD8- cells (C), CD3-CD8- cells (D) and CD3+CD8+ T cells (E). In panels C, D and E the upper right quadrant shows PD-1+GrzB+ cells, the upper left quadrant shows PD-1+GrzB- cells, the lower right quadrant shows PD1-GrzB+ cells and the lower left quadrant shows PD-1-GrzB- cells. The staining panel comprised anti-CD3 PERCP, anti-CD8 FITC, anti-PD-1 PE and Granzyme B APC. Isotype controls were used for PD-1 and Granzyme B and Fluorescence Minus One Controls (FMOs) were used to set the gates accordingly. (TIF) [file pone.0137646.s001.tif]
